# Tuning the instrument resolution using chopper and time of flight at the small-angle neutron scattering diffractometer KWS-2

**DOI:** 10.1107/S1600576715019019

**Published:** 2015-11-19

**Authors:** Aurel Radulescu, Noémi Kinga Székely, Stephan Polachowski, Marko Leyendecker, Matthias Amann, Johan Buitenhuis, Matthias Drochner, Ralf Engels, Romuald Hanslik, Günter Kemmerling, Peter Lindner, Aristeidis Papagiannopoulos, Vitaliy Pipich, Lutz Willner, Henrich Frielinghaus, Dieter Richter

**Affiliations:** a Jülich Centre for Neutron Science (JCNS), Outstation at MLZ, Forschungszentrum Jülich GmbH, Lichtenbergstrasse 1, Garching, 85747, Germany; b Zentralinstitut für Engineering, Elektronik und Analytik (ZEA), Forschungszentrum Jülich GmbH, Jülich, 52425, Germany; c Jülich Centre for Neutron Science (JCNS) and Institute for Complex Systems (ICS), Forschungszentrum Jülich GmbH, Jülich, 52425, Germany; d Institute for Complex Systems (ICS), Forschungszentrum Jülich GmbH, Jülich, 52425, Germany; e Institut Laue–Langevin, 71 Avenue des Martyrs, Grenoble, 38000, France; f Theoretical and Physical Chemistry Institute, National Hellenic Research Foundation, 48 Vass. Constantinou Avenue, Athens, 11635, Greece

**Keywords:** small-angle neutron scattering (SANS), time of flight, wavelength spread

## Abstract

Following demand from the user community regarding the possibility of improving the experimental resolution, the dedicated high-intensity/extended *Q*-range SANS diffractometer KWS-2 of the Jülich Centre for Neutron Science at the Heinz Maier-Leibnitz Center in Garching was equipped with a double-disc chopper with a variable opening slit window and time-of-flight (TOF) data acquisition option. The chopper used in concert with a dedicated high-intensity velocity selector enables the tuning at will of the wavelength resolution Δλ/λ within a broad range, from 20% (standard) down to 2%, in a convenient and safe manner following pre-planned or spontaneous decisions during the experiment. The new working mode is described in detail, and its efficiency is demonstrated on several standard samples with known properties and on a completely new crystallizable copolymer system, which were investigated using both the conventional (static) and TOF modes.

## Abstract 2

Using a double-disc chopper with a variable slit opening in concert with a velocity selector and the time-of-flight data acquisition mode, controlled variation of the wavelength spread Δλ/λ between 2 and 20% has become routinely possible at the KWS-2 SANS diffractometer of the Jülich Centre for Neutron Science at the Heinz Maier-Leibnitz Center.

## 1. Introduction   

The small-angle neutron scattering (SANS) diffractometer KWS-2 was reconstructed by the Jülich Centre for Neutron Science (JCNS) at the neutron source Heinz Maier-Leibnitz (FRM II reactor) in Garching (Gläser & Petry, 2000 ▸) in several steps. The reconstruction process was completed during the long reactor shutdown in 2011, when the instrument reached its nominal configuration of a 42 m-long classical pinhole SANS diffractometer, as used at the DIDO reactor in Jülich (Schwahn *et al.*, 1991 ▸). The conventional setup of KWS-2 permits the exploration of a wide range of momentum transfers *Q* by variation of the sample-to-detector distance between 1 and 20 m, and by variation of the wavelength between 4.5 and 20 Å. In contrast to the previous setup in Jülich, where structural investigations on the length scale between 10 and 500 Å have been typically performed at the neutron wavelength λ = 7 Å, the much higher flux at the FRM II and the dedicated neutron guide system (Radulescu & Ioffe, 2008 ▸; Radulescu, Pipich & Ioffe, 2012 ▸) allow the use of longer wavelengths, up to 20 Å, thus enabling much lower values in *Q*, down to 7 × 10^−4^ Å^−1^.

At the same time, following demand from the user community, KWS-2 was considerably upgraded, aiming to boost its performance with respect to the three figures of merit of an instrument of this kind, namely the resolution function, the intensity on the sample and the minimum scattering variable (*Q*_min_). For this purpose, the instrument was equipped with a double-disc chopper with a variable slit opening, focusing elements [MgF_2_ parabolic lenses, as reported by Frielinghaus *et al.* (2009 ▸)] and a secondary high-resolution position-sensitive detector (scintillation, 1 mm position resolution and 0.45 mm pixel size, placed at 17 m after the sample and vertically movable in out-of-beam–in-beam positions). Besides the conventional pinhole measurement mode, the following setups were tested in 2013 and became routinely available for experiments in 2014, allowing for an increased instrument flexibility and versatility: (i) the tunable resolution mode enables controlled variation of the wavelength spread Δλ/λ between 2 and 20% and thus permits an improved characterization of the scattering features within different *Q* ranges by using the chopper and the time-of-flight (TOF) data acquisition method; (ii) the high-intensity mode with lenses yields an intensity gain of up to 11 times compared to the conventional pinhole mode for the same resolution owing to an increase of the sample size (up to 5 cm in diameter); (iii) the high-resolution/extended *Q*-range mode, by means of the lenses in combination with the chopper (for narrowing Δλ/λ and minimizing the chromatic aberrations and gravity effects) and the high-resolution detector, enables one to decrease *Q*_min_ down to 1 × 10^−4^ Å^−1^. Combined with the conventional pinhole this allows for the exploration of a continuous length scale from 1 nm to 1 µm in an easy manner at the same instrument. This approach becomes attractive when the amount of sample is insufficient and when the sample is exposed to irreversible transformations in external fields (temperature, pressure *etc*.), so that the characterization over a wide length scale by combining different instruments is impossible.

General details about the standard configuration and parameters of the KWS-2 SANS diffractometer were presented in earlier publications (Radulescu, Pipich & Ioffe, 2012 ▸; Radulescu, Pipich, Frielinghaus *et al.*, 2012 ▸). In this paper we present in detail the new working mode that enables the tuning of instrumental resolution using the chopper and TOF data acquisition. Preliminary results obtained during the commissioning of this mode are shown in parallel and in combination with the conventional pinhole mode, and the advantages and gains in performance by using this method are discussed. The other two major upgrades – the high-intensity mode and the extended *Q*-range mode using focusing lenses – are the subject of a forthcoming paper.

## 2. SANS resolution   

The typical large wavelength spread Δλ/λ and the limited geometric resolution of a SANS instrument (finite collimation, detector resolution, gravity) lead to smearing that can cause losses of the structural information contained in the SANS curve. The resolution of a SANS instrument can, in principle, be modelled by a Gaussian function of the standard deviation σ_*Q*_ of the scattering variable *Q*, where *Q* = 4π sin(θ)/λ with λ the neutron wavelength and 2θ the scattering angle. A comprehensive discussion of the SANS resolution can be found in the work of Mildner & Carpenter (1984 ▸), Pedersen *et al.* (1990 ▸) and Hammouda & Mildner (2007 ▸). Here, we only briefly present the notions which are important for understanding how the tuning of the resolution is currently achieved at KWS-2.

In general, for the pinhole SANS geometry the standard deviation σ*_Q_* consists of two contributions:
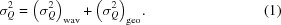
The first term is the *Q*-dependent wavelength spread contribution and is given by
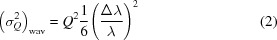
in the case of a triangular wavelength distribution of full width at half-maximum (FWHM) Δλ. The second term represents the ‘geometry’ contribution:
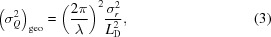
which depends on the instrumental configuration (the size of the source *R*_E_ and sample *R*_S_ apertures, the collimation length *L*_C_ and the sample-to-detector distance *L*_D_) and the detector resolution through the horizontal and vertical components of the spatial variance 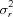 (Hammouda & Mildner, 2007 ▸). The vertical component contains an additional contribution owing to the gravity effect on the neutron trajectory, which, besides the collimation length and the sample-to-detector distance, depends on the fourth power of λ and the second power of Δλ/λ (Hammouda & Mildner, 2007 ▸).

**Figure 1:**
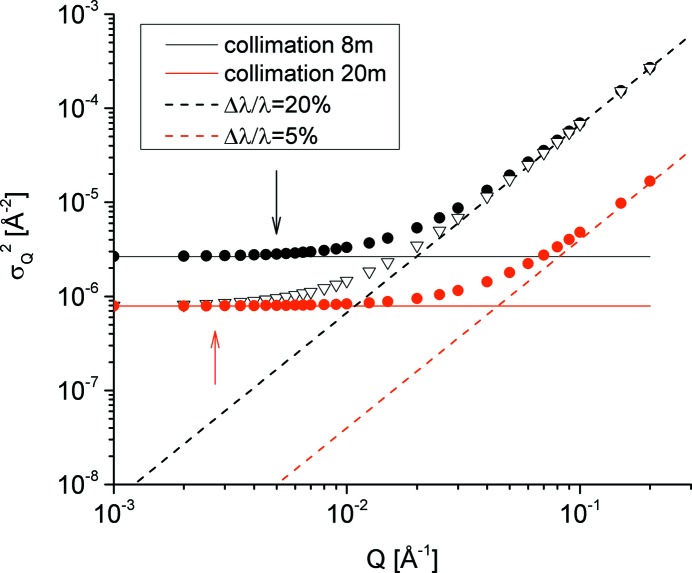
The wavelength spread (dashed lines) and ‘geometry’ (solid lines) contributions to the total *Q* distribution 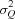 (symbols) for some specific experimental configurations at KWS-2; the curves are calculated for λ = 5 Å, *L*_D_ = 8 m, *R*_E_ = 16.9 mm, *R*_S_ = 5.64 mm and for two setups of collimation length (*L*_C_ = 8 and 20 m) and wavelength spread (Δλ/λ = 20 and 5%). The arrows indicate the *Q*_min_ reached for the considered configurations (for *L*_C_ = 20 m the *Q*_min_ values for the two different wavelength spreads are very close to each other – see Table S1).

Fig. 1 ▸ presents the wavelength spread and geometry contributions to the total *Q* distribution 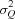 as a function of *Q* for different experimental configurations at KWS-2. The geometrical contribution is constant *versus Q* and dominates at low *Q*, whereas the contribution from Δλ/λ is *Q* dependent and plays an important role towards large *Q* values. As can be observed in this example, the resolution of a SANS instrument (symbols in Fig. 1 ▸) can thus be adjusted on demand by varying the wavelength spread Δλ/λ and/or geometrical configuration within certain limits imposed by the technical equipment of the instrument. The greatest improvement in resolution can be achieved when both the ‘geometric’ and the wavelength spread contributions are simultaneously minimized (red dots in Fig. 1 ▸). It should also be noted that there is always a matter of compromise between intensity and resolution and that any improvement in resolution is made at the cost of intensity on the sample.

Another quantity that can be adjusted by varying the wavelength spread Δλ/λ or geometry of the instrument is *Q*_min_. Again, a detailed discussion on this quantity can be found in the paper by Hammouda & Mildner (2007 ▸). Although this figure of merit of the KWS-2 SANS diffractometer will be presented in detail in a forthcoming paper together with the working modes employing focusing lenses, here we will briefly present the consequences of the adjustments of the standard deviation 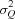 of the SANS resolution on this quantity. The minimum values of *Q* which can be reached for the instrument configurations described in Fig. 1 ▸ are indicated by the arrows. For SANS experiments performed in pinhole geometry *Q*_min_ is in practice determined by the neutron spot size in the vertical direction *Y*_min_ where the beam is broadest owing to the effect of gravity on the neutron trajectories:
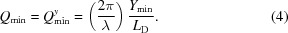
Typically, an asymmetric oval beam spot on the detector is obtained, whose shape and size in the vertical direction are dictated by the instrument geometry and depend on the second power of the neutron wavelength λ and on the wavelength distribution Δλ/λ. In Table S1 (see the supporting information) the variance 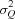 of the *Q* resolution, the beam spot size *X*_min_ and *Y*_min_ in both horizontal and vertical directions, and 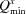 and 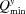 are reported for several instrument configurations, as obtained by following the approach described by Hammouda & Mildner (2007 ▸). For neutron wavelengths typically used for SANS experiments, between 4.5 and 7 Å, the gravity effects are small even for long sample-to-detector distances or broad wavelength distributions. At long wavelengths the effect is very pronounced and hinders the measurements and data analysis for a wavelength spread Δλ/λ within the range 10–20% that is used by most SANS instruments. This becomes a problem especially in the case of using focusing lenses for pushing *Q*_min_ towards lower values. Besides the distortion in the vertical direction due to gravity, additional effects due to chromatic aberration occur in both horizontal and vertical directions in this case (Hammouda & Mildner, 2007 ▸; Frielinghaus *et al.*, 2009 ▸). In order to reduce these effects the wavelength spread Δλ/λ has to be minimized.

## 3. Wavelength spread resolution Δλ/λ at KWS-2   

For a pinhole SANS diffractometer the adjustment of the geometry contribution to the *Q* resolution can be achieved by changing the collimation conditions of the incoming beam. This implies the variation of collimation length, as shown in Fig. 1 ▸, and/or the variation of the aperture size. Since KWS-2 is equipped with variable collimation systems characterized by high versatility and automated selection of the source-to-sample distance and aperture sizes, this adjustment can be made routinely without requiring complicated technical changes during the experiment. The spatial detector resolution for a conventional SANS instrument lies typically between 5 and 8 mm and, unless the instrument is equipped with several detectors with different cell size, it is thus a fixed instrumental parameter.

Unlike the variation of the geometric term of the resolution, the adjustment of the wavelength resolution is more difficult to accomplish practically. A sufficiently high neutron flux on the sample demands in the case of a conventional SANS instrument a broad wavelength spread Δλ. Such beams are usually obtained by using mechanical monochromators (velocity selectors). These are robust drum-type devices which operate in transmission geometry and exist in either multiple discs with windows (Friedrich *et al.*, 1989 ▸) or helical channels (Hammouda, 1992 ▸) configurations rotating about an axis parallel to the beam direction. The neutron wavelength λ is determined by the rotation speed, and a fixed resolution Δλ/λ, typically within the range from 5 to 40%, is achieved for a fixed geometry of the device. The tilting of the rotation axis of the selector with respect to the beam axis determines the transmitted wavelength λ and resolution Δλ/λ (Wagner *et al.*, 1992 ▸). Thus, for positive tilting angles the transmitted wavelength λ is increased and the resolution Δλ/λ is improved, while for negative tilting angles the wavelength λ is decreased and the resolution is relaxed (here, a positive angle corresponds to a tilting towards the screw direction of the helical path of the selector). 

Nevertheless, this option offers reduced possibilities for adjusting the resolution of a selector and is always accompanied by a simultaneous change in wavelength, which makes it not very attractive for routine scientific applications. For example, the specifications of velocity selectors produced by Astrium GmbH indicate that a device which is designed with mechanical characteristics for delivering a wavelength λ = 4.5 Å with Δλ/λ = 10% in the standard configuration (parallel to the beam axis) would deliver λ = 2.5 Å with Δλ/λ = 20.6% and λ = 6.4 Å with Δλ/λ = 8.2% at tilt angles of −10 and 10°, respectively. Thus, in this way only a modest improvement in resolution can be obtained at high technical cost, since the tilting of a velocity selector during an experiment is time consuming and implies severe safety considerations. A strongly improved or relaxed nominal wavelength resolution can be achieved using dedicated selectors, designed and constructed for fulfilling the customer’s specific demands. In this case, the transmitting window between the absorbing regions of the selector can be purposely increased (poorer resolution/high intensity) or decreased (better resolution/low intensity). This basically means a variation of the number of tilted blades or windows defining the helical path for neutrons. Most SANS instruments use selectors that provide a fixed Δλ/λ of about 10%, with narrow possibilities to vary it by tilting, which offers a resolution/intensity balance fulfilling most users’ common requests. To our knowledge, a couple of SANS instruments are already (Gilbert *et al.*, 2006 ▸) or will be (Gilles *et al.*, 2007 ▸) equipped with an additional selector enabling Δλ/λ = 5.4–6% while operating parallel to the beam axis. Although delivering a beam with improved wavelength resolution, these selectors have nevertheless the disadvantage that they have to operate with lower rotation speed because of their increased weight and can therefore deliver only wavelengths λ > 6.4 Å (Gilles *et al.*, 2007 ▸). Tilting such selectors under certain angles for delivering neutrons of shorter wavelength will drastically worsen the Δλ/λ resolution. On the other hand, the operation of two or more selectors in turn for different resolutions requires a huge technical effort and special care. The high alignment precision of the beam, stability and safety while operating in high vacuum, the high rotation frequencies, and the need for large casemates as radiation shielding are implied in this case. Although this concept is an attractive method, it nevertheless has drawbacks in terms of cost, safety in operation, ease of handling and its practicability for an intensive user program like that held at KWS-2.

**Figure 2:**
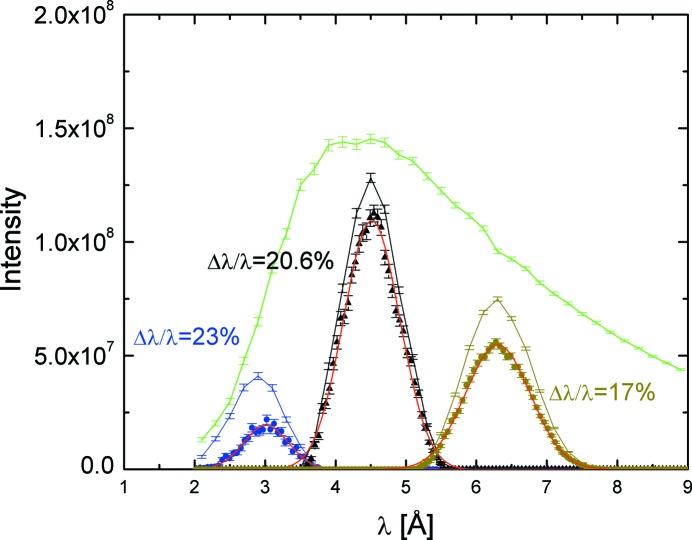
Wavelength distribution in the neutron guide system of KWS-2 simulated using *McStas*, showing the white beam before the velocity selector (green line) and the beam after the velocity selector rotating at a speed of 28 200 r min^−1^ and with different tilt angles (blue: −10°; black: 0°; brown: +10°); the symbols represent simulations at the end of the ‘S-shaped’ guide, for the same tilting angles; the red lines denote the fits of the Gaussian distributions which delivered the Δλ/λ for the three setups. The transmission factors for the three different wavelengths can be directly determined from the figure.

The large demand for high-intensity investigations at the JCNS SANS diffractometers led to an early decision to equip the KWS-2 instrument with a velocity selector that typically enables a relaxed resolution, Δλ/λ = 20%. The beam and velocity selector characterization at KWS-2 has been reported elsewhere (Radulescu & Ioffe, 2008 ▸; Radulescu, Pipich & Ioffe, 2012 ▸). Owing to geometrical constraints and following detailed simulations by *McStas* (Willendrup *et al.*, 2004 ▸) for the optimization of the instrument neutron guide, it was decided to place the velocity selector in the middle of the vertically ‘S-shaped’ guide in a slightly inclined fixed position, in order to have its axis parallel with the beam axis. Fig. 2 ▸ displays the beam intensity distribution at different positions along the neutron guide system as determined by *McStas* simulations using a realistic model for the cold neutron source of the FRM II reactor (Zeitelhack *et al.*, 2006 ▸) and the real parameterization of the neutron guide system upstream of KWS-2. In front of the selector the ‘white beam’ shows a broad wavelength distribution with a cut-off slightly below 2.5 Å, which is the result of the curved guides installed up to this position. The three upper peak-like features (lines) indicate the intensity distribution of the monochromatic beam after the velocity selector (Astrium GmbH, with 36 ^10^B-coated carbon fibre blades) for the selector running with a speed of 28 200 r min^−1^ and tilted 10° (left), 0° (middle) and −10° (right) with respect to the beam axis. The lower peak-like features (symbols) represent the intensity distribution at the end of the vertically ‘S-shaped’ guide (instrument entrance) for the same selector configurations. By fitting the Gaussian shaped intensity distribution at the end of the S-guide and interpreting the FWHM one can see that tilting of the selector by +10° with respect to the beam axis would provide a very modest improvement of the Δλ/λ resolution. Therefore, this option was dismissed as a possibility for the adjustment of wavelength resolution at KWS-2 from the beginning. On the other hand, the selector tilting over an angle of −10° is scientifically more interesting owing to the possibility of achieving lower wavelengths, down to λ = 3 Å, with significant intensity. In this case, for the shortest collimation length and thus the low-resolution–high-*Q* instrument operation mode, the simulated neutron flux at the sample position is 3 × 10^7^ n cm^−2^ s^−1^. It is planned to explore this opportunity practically at KWS-2 in the near future.

**Figure 3:**
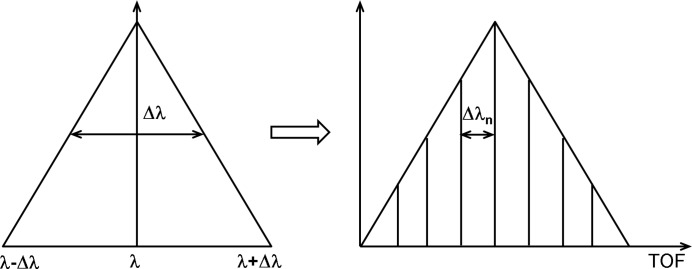
Schematic representation of the principle of tuning the wavelength resolution at KWS-2: the triangular wavelength distribution delivered by the velocity selector is decomposed on the detector into several TOF channels using a chopper with variable slit opening and rotation frequency; each channel features a narrower wavelength distribution.

Although the instrument is consistently overbooked and the high-intensity/relaxed-resolution conventional mode is in high demand, there are increasing calls for improved resolution, following either pre-planned or spontaneous decisions, during the experiments. Requests from the fields of crystallizing polymers or biomacromolecules would especially benefit from the flexibility of tuning the resolution Δλ/λ within a broad range, from 20% down to 2 or 1%. Such an increased degree of variability accompanied by convenient and safe handling is currently achieved at KWS-2 by using a double-disc chopper with a variable slit opening in concert with the selector. In this case, a pulsed beam is generated and the data acquisition on the detector is carried out using the TOF technique. The wavelength band delivered by the selector Δλ can be divided into *n* equal parts of a narrower Δλ_*n*_ (Fig. 3 ▸), where a desired value of *n*, and thus of Δλ_*n*_, can be achieved by the appropriate adjustment of the width of the slit opening and rotation frequency of the chopper with the neutron wavelength λ and detection distance *L*_D_.

The operation mode of the chopper as well as the procedure for selecting the resolution and its verification using standard samples is presented in the following. The design and construction details are reported in the supporting information.

## 4. The selection of wavelength resolution   

The resolution and intensity aspects of single- and double-disc chopper systems are discussed in detail by Copley (1990 ▸), van Well (1992 ▸) and van Well & Fredrikze (2005 ▸). In TOF mode the wavelength resolution Δλ/λ is given by the time *t* a neutron with wavelength λ needs to travel the distance from the chopper to detector, which is convoluted with the time spread τ of the pulsed beam at the source position. The KWS-2 chopper may be practically considered a single-disc chopper as the TOF resolution is dictated mainly by the opening time of the chopper window τ_w_, which depends on the chopper frequency and angular opening of the window, Δφ. For a specific chopper/instrument configuration the wavelength resolution Δλ/λ is inversely proportional to λ. The resolution can be improved if the rotation frequency is increased, the window opening is decreased or the total flight time is increased. The transmission of the single-disc chopper depends only on its construction characteristics.

**Figure 4:**
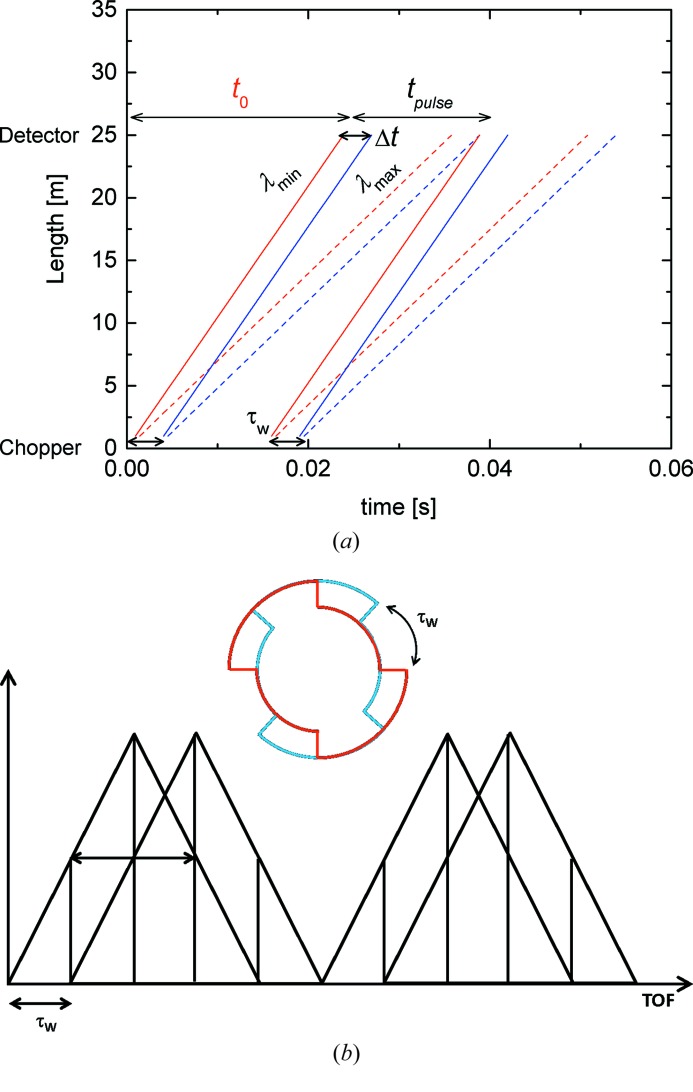
The TOF diagram (*a*) and a schematic presentation of the time distribution and splitting of the double pulse emitted per cycle and collected within the TOF window set for the detector (*b*) for the setup corresponding to Δλ/λ_aim_ = 10%: *L*_D_ = 4 m, λ = 4.72 Å, *f*_chopper_ = 34 Hz, Δφ = 36°; τ_w_ = 0.0029 s. To reach the Δλ/λ_aim_ each pulse must be split into five TOF slots. The two partially overlapping triangles which are shown in (*b*) represent the distribution of neutrons from the opening and closing edges of the chopper.

In the simple TOF mode of the KWS-2 chopper one aims to decompose the wavelength band of the selector into *n* equal parts (as schematically shown in Fig. 3 ▸). For this, in principle the opening time of the chopper window τ_w_ is set to match the *n*th part of the wavelength band. Figs. 4 ▸ and 5 ▸ present for two routinely used experimental configurations the time–distance diagrams and schematically the principle of decomposing the wavelength band recorded on the main detector (Kemmerling *et al.*, 2004 ▸) for two particular improvements in Δλ/λ. The chopper transforms the continuous incoming beam into a pulsed beam, emitting two pulses with time width τ_w_ at each rotation. The pulse time width widens as neutrons with the wavelength distribution Δλ/λ propagate to the detector, where they are recorded as a function of the flight time. The TOF data acquisition window of the detector electronics is initialized once per chopper rotation (always when the pickup magnet of the master disc of the chopper passes the pickup sensor – see Fig. S3 in the supporting information), and thus two spread pulses are always recorded on the detector according to their arrival time. A certain time delay between the issuing of the trigger signal and the opening of the neutron guide is involved, which is always known considering the chopper settings and instrument configuration. Also, there is a time *t*_0_ between the initialization of the TOF acquisition window and the arrival of the fastest neutrons (λ_min_) of the first pulse on the detector. This time *t*_0_ depends on the experimental configuration (λ_min_ and *L*_D_) and should be taken into account when the TOF scale is converted into wavelength scale. 

The repetition rate relates to the spread at the detector position of the two pulses emitted for each chopper rotation and must be adjusted to the wavelength band 2Δλ delivered by the selector. The detection window for the spread in time between two master pulses consists of the standard configuration of 64 TOF channels, but the number can be adjusted at will. The width of the window (and thus that of a TOF channel) is adjusted as a function of chopper settings and experimental conditions in order to avoid overlapping between the two pulses emitted per cycle or between pulses belonging to adjacent cycles.

A full single pulse recorded on the detector will thus have a total spread *t*_pulse_ corresponding to the total time of (*n* + 1)τ_w_. In an optimal computational and data processing approach the channels of the TOF scale are grouped so that the final data are delivered on time slots with a width Δ*t* = τ_w_ (Figs. 4 ▸ *a* and 5 ▸ *a*). With the spread of neutron wavelength (FWHM of an assumed Gaussian distribution) in each time slot *i* Δλ_*i*_ = Δλ/*n* (where *i* goes from 1 to *n*), the wavelength resolution is then Δλ_*i*_/λ_*i*_, with λ_*i*_ the mean wavelength corresponding to each slot.

By matching the chopper frequency and slit opening with the *L*_TOF_ distance and wavelength band delivered by the selector, the resolution can be adjusted to any desired value, in principle. The frequency range from 25 to 32 Hz needs to be avoided because of resonance effects.

The single pulse spread on the detector is defined by
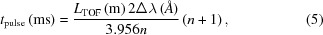
where *L*_TOF_ = 0.7 m + 20 m + *L*_D_ is the chopper-to-detector distance and 2Δλ is the wavelength band delivered by the selector, which at KWS-2 is 2Δλ = 2(Δλ/λ)λ = 0.4λ.

Considering the particular cases presented in Figs. 4 ▸ and 5 ▸, with an initial wavelength distribution Δλ/λ = 20%, as delivered by the velocity selector, each pulse is divided into *n* + 1 = 5 time slots, in the case Δλ/λ_aim_ = 10%, while for Δλ/λ_aim_ = 5% *n* + 1 = 9 time slots are needed. In order to avoid the overlapping of pulses and to achieve the targeted wavelength resolution, the chopper frequency and the slit opening were set at *f*_chopper_ = 34 Hz, Δφ = 36° and *f*_chopper_ = 32.4 Hz, Δφ = 20°, respectively. One should also note that the targeted resolution Δλ/λ_aim_ is ideally obtained only in the central time slot, with the resolution in the earlier/later slots being slightly worse/better (van Well & Fredrikze, 2005 ▸). The control software of the KWS-2 instrument enables increased flexibility and variability during an experimental session, enabling the combination of the conventional (continuous) and TOF measurement modes for different wavelengths λ and collimation/detection distances *L*_C_/*L*_D_. Also, an automatic setting of chopper parameters is possible for achieving different wavelength resolutions between 2 and 10% at will for different selected λ and *L*_D_.

**Figure 6:**
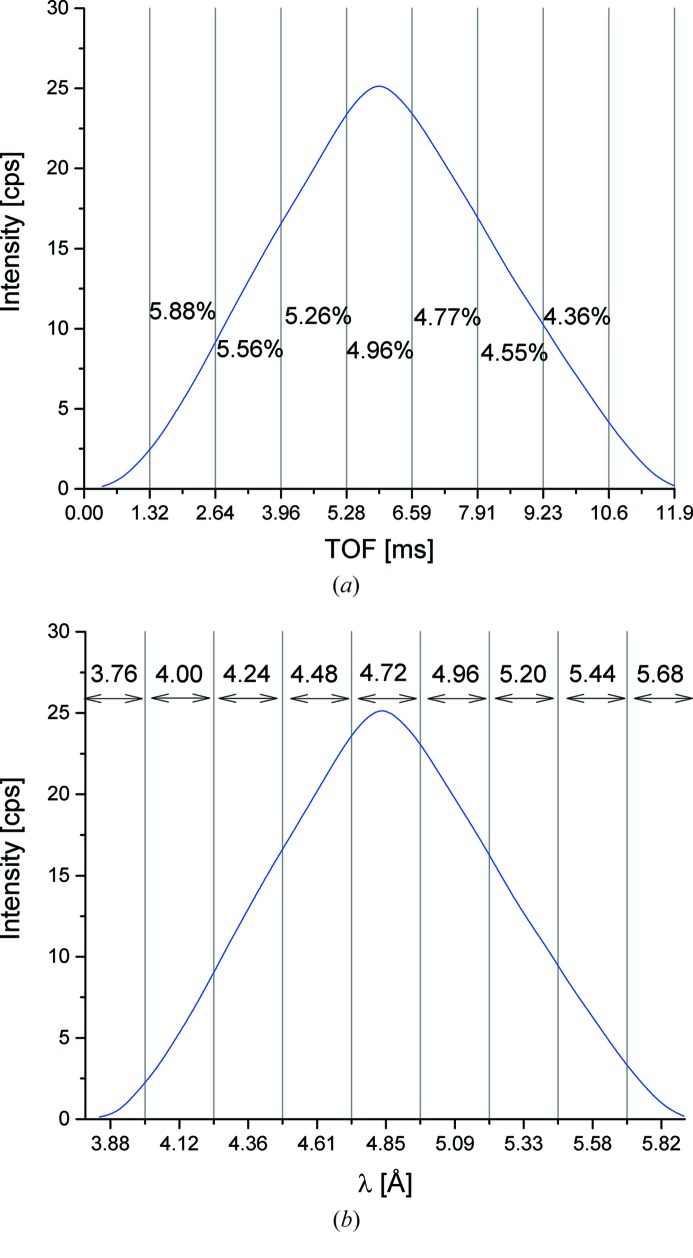
Measured direct beam intensity on the detector (*L*_D_ = 1.3 m) for λ = 4.72 Å as a function of TOF (*a*) and of recalculated wavelength (*b*) according to the measured TOF and experimental settings corresponding to Δλ/λ_aim_ = 5%; the real Δλ/λ calculated from the measured *t* and Δ*t* corresponding to each time slot is indicated in (*a*); the mean wavelength of each slot is indicated in (*b*), as defined by the left flank of the slot, according to the scheme shown in Fig. 5 ▸(*b*).

The data analysis software *QtiKWS* (http://iffwww.iff.kfa-juelich.de/~pipich/dokuwiki/doku.php/qtikws) enables the further processing of data: the correction for the time delay between issuing the master pulse and the opening of the guide, the merging of the two similar pulses recorded in the TOF window, the conversion to real time scale (Fig. 6 ▸), the splitting into an appropriate number of time slots for reaching the Δλ/λ_aim_ (Fig. 6 ▸ *a*) and the conversion of time scale into wavelength scale (Fig. 6 ▸ *b*). Finally, the typical SANS data reduction procedure is applied to the data: the correction for empty beam or empty cell, the detector sensitivity and electronic noise, and the calibration in absolute units using standard samples (Radulescu *et al.*, 2012 ▸). All correction and calibration measurements are carried out in TOF mode.

The recalculation of the wavelength (Fig. 6 ▸ *b*) after merging the two similar pulses is done from the measured TOF (Fig. 6 ▸ *a*) according to
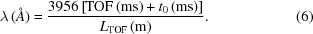
As can be understood from the schemes in Figs. 4 ▸(*b*) and 5 ▸(*b*), where the two triangles shown for each pulse define the distribution of neutron wavelengths from the opening and closing edges of the chopper window, the mean wavelength of each time slot in Fig. 6 ▸(*b*) is in practice given by the recalculated wavelength at the left edge of the slot. The resolution in each time slot is calculated according to Δλ/λ = τ_w_/(TOF + *t*_0_), where TOF is the time of each slot in Fig. 6 ▸(*a*).

**Figure 7:**
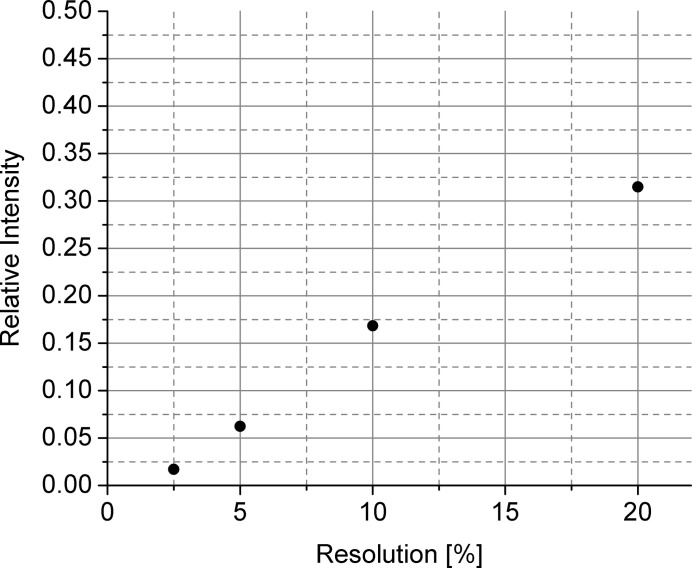
The intensity provided by the tandem selector–chopper relative to that delivered by the selector for different wavelength resolutions.

Fig. 7 ▸ presents the intensity provided by the combination of selector–chopper relative to that delivered by the selector for different wavelength resolutions, as emerging from direct beam measurements for λ = 4.72 Å and *L*_C_ = 20 m (the detector was placed at *L*_D_ = 4 m). It can be observed that, although a significant intensity decrease occurs for a resolution improvement of 4–8 times compared to the standard configuration (using only the selector), there is sufficient intensity available for performing reliable experiments with wavelength resolutions at wavelengths for which no dedicated velocity selectors exist. Also, almost no useful intensity was experimentally observed for Δλ/λ < 2%, which is a consequence of the chopper disc geometry (design and opening).

It is also worth adding that inelastic scattering effects occurring in the case of incoherently scattering hydrogenated samples (Ghosh & Rennie, 1990 ▸; Rennie & Heenan, 1993 ▸; Do *et al.*, 2014 ▸) induce a shift in neutron energy from longer to shorter wavelength. Consequently a large portion of neutrons are scattered by the sample with a gain in energy that corresponds to a wavelength distribution centred at around 1.5 Å (Ghosh & Rennie, 1990 ▸). The inelastic scattering from such materials becomes most significant as the incident wavelength increases. This effect results in the deterioration of the typical pulse distribution observed on the TOF scale (Fig. S8) and makes the analysis of the data collected at longer detection distances (*L*_D_ > 2 m) for wavelengths λ > 7 Å difficult. This case, together with results of the tests of various soft-matter samples with different amounts of hydrogen, will be presented in detail in a forthcoming paper. On the other hand, the technique will be beneficial for a large number of hard-matter and nanostructured systems that would not exhibit significant inelastic scattering.

Finally, preliminary tests have demonstrated that using the chopper for recording data in TOF mode with an improved resolution (Δλ/λ < 5%) may be of great advantage for high-resolution measurements using lenses (with small entrance aperture at *L*_C_ = 20 m) and a secondary high-resolution detector with long wavelengths, towards λ = 18–20 Å, when otherwise the gravity effect would spoil the results for Δλ/λ = 20%. In this case, instead of a beam spot which is very narrow in the horizontal direction (owing to focusing), but broad in the vertical direction (owing to gravity), several round-shaped symmetric beam spots would be detected at different times on the TOF scale by splitting the initial broad wavelength distribution (Δλ/λ = 20%) into many narrower distributions. These narrow and isotropic beams (weak out-of-focus effects affect the marginal wavelengths of the broad distribution) show a vertical distribution of their position, as a function of λ. Another advantage in terms of transmission and homogeneity of the cooling (lenses are used at the temperature *T* = 70 K) is offered by the fact that for this setup only four lenses are needed for the fixed measurement geometry, *L*_D_ = 17 m (Frielinghaus *et al.*, 2009 ▸). The problem with the drop in intensity due to the use of the chopper may be minimized by using larger samples, up to 5 cm in diameter. As for the inelastic/incoherent case the high-resolution/gravity effects will be reported in detail in a forthcoming paper.

## 5. Application to experimental data   

As examples of application of the method to real experimental data, scattering experiments on several standard systems and one new diblock copolymer system of scientific interest were chosen.

As standards typically used for the calibration of the wavelength and wavelength spread in SANS, the following systems were tested: (*a*) SiO_2_ particles in deuterated dimethyl-formamide (d-DMF) as size standards with a particle volume fraction of 0.2% and well known size and polydispersity (Vad *et al.*, 2010 ▸); (*b*) silver behenate (AgBeh) as a wavelength and wavelength spread standard (Huang *et al.*, 1993 ▸; Gilles *et al.*, 2000 ▸; Okabe *et al.*, 2007 ▸); (*c*) opal (Graetsch & Ibel, 1997 ▸) as a wavelength standard. Star-like micelles formed by newly synthesized C_28_H_57_–PEO5 diblock copolymers in water (Zinn *et al.*, 2014 ▸) were for the first time investigated by SANS with variable wavelength resolution in order to scan the crystalline phases occurring over a wide polymer concentration range.

### 5.1. Size standard SiO_2_ particles   

**Figure 8:**
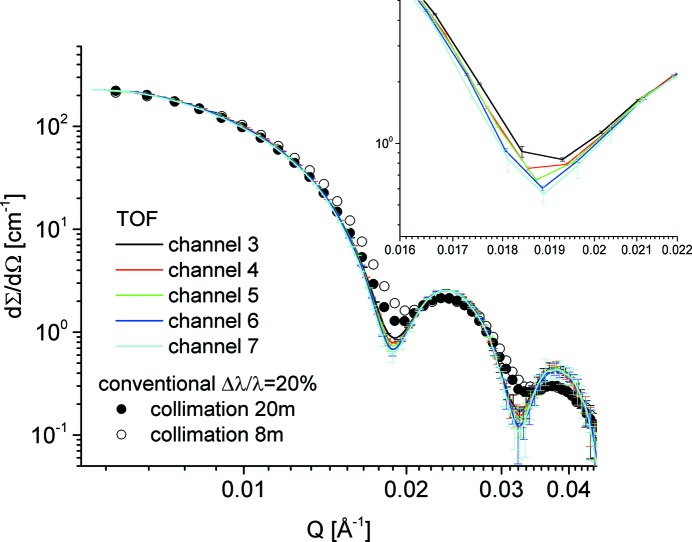
SANS curves of SiO_2_ particles in d-DMF measured at KWS-2 with different resolutions; symbols – data collected with the conventional (continuous) mode by varying the collimation length *L*_C_; lines – data collected with the TOF mode using the chopper adjusted for Δλ/λ_aim_ = 5% (see details in text) as recorded in different time slots (channels). The inset shows the first minimum in the form factor.

The structure parameters of SiO_2_ particles in d-DMF are well known from previous small-angle X-ray scattering (SAXS) and SANS experiments (Vad *et al.*, 2010 ▸). The structure parameters characterizing the particles that are important for the present test are the radius *R*_0_ = 241.3 Å and the polydispersity parameter σ_0_ = 0.0323. For the particle volume fraction that was measured, no structure-factor effects were observed in the low-*Q* scattering curves. The scattering cross section for SiO_2_ particles measured at the detection distance *L*_D_ = 8 m with λ = 4.72 Å is shown in Fig. 8 ▸ for the different instrumental resolution conditions used. By improving the ‘geometrical resolution’ by increasing the collimation length from *L*_C_ = 8 m to *L*_C_ = 20 m (Table S1) and the ‘wavelength resolution’ from Δλ/λ = 20% to Δλ/λ = 5% by using the chopper and TOF data acquisition, the form-factor features (minima and maxima) towards high *Q* become better defined, which will enable a more accurate fit using the spherical form-factor model function convoluted with the instrumental resolution. The chopper was used at a frequency *f* = 32.4 Hz with an opening slit window of Δφ = 20°. The TOF data were analysed using the procedure described above in §4 using nine TOF slots (channels). From the inset of Fig. 8 ▸, where the first form-factor minimum as collected in the time channels from 3 to 7 is shown, it can be observed that the resolution varies weakly from one channel to another. As mentioned before, the targeted wavelength spread Δλ/λ_aim_ = 5% is expected to be achieved in the middle channel, No. 5.

Knowing beforehand the structural parameters of the disperse SiO_2_ particles, an attempt was made to determine the wavelength resolution term from the model fit of the data. The SANS data were fitted by a form factor of polydisperse spheres (Pedersen, 1997 ▸), where the effect of polydispersity was taken into account by a Gaussian distribution function,
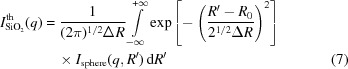
with
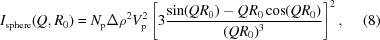
where *R*_0_ and σ_0_ = Δ*R*/*R*_0_ are known from previous SAXS and SANS measurements. The number density of particles *N*_p_, neutron scattering contrast Δρ and particle volume *V*_p_ were incorporated in a single fitting parameter, *p* = *N*_p_Δρ^2^ *V*_p_^2^, which does not affect the shape and characteristic features of the SANS curves, *i.e.* is not relevant to determining the instrument’s resolution function.

The theoretically modelled intensities 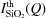 are convoluted (Barker & Pedersen, 1995 ▸) with a Gaussian function so that the instrumental smearing σ_*Q*_(*Q*) defined in equation (1) is taken into account:
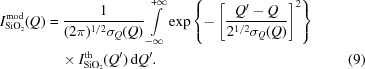



The convoluted curves 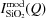 are the ones that can be compared with and consequently model the experimental data. The integration in equation (9) is adequately performed by Simpson’s rule.

**Figure 9:**
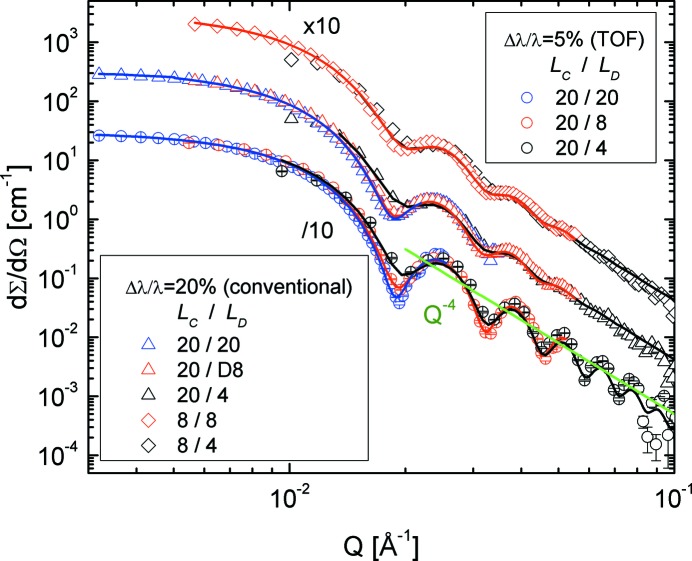
SANS curves of the SiO_2_ particles in d-DMF measured at KWS-2 with different configurations and model intensities according to equation (9). The triangles and lozenges depict data collected with the standard continuous mode (different *L*_C_–*L*_D_ configurations), while the circles represent data collected with TOF mode using the chopper (adjusted for Δλ/λ_aim_ = 5%) as recorded in the central time slot (channel 5). The power-law behaviour towards high *Q* is also indicated.

Data sets obtained under different conditions (wavelength spread and instrument configurations) are shown in Fig. 9 ▸. The experimental curves were fitted simultaneously with fixed parameters *R*_0_, σ_0_ and σ_geo_ [calculated for each configuration according to equation (3), see Table S1] and Δλ/λ as fitting parameter for each configuration. The model curves in Fig. 9 ▸ describe very well the experimental data and delivered for the two sets of configurations (conventional SANS and TOF) a wavelength spread of Δλ/λ = 19.8% and Δλ/λ = 5.3%, respectively. In the case of the TOF measurement the result is very close to the expected wavelength spread Δλ/λ_aim_.

### 5.2. Wavelength standard silver behenate (AgBeh)   

The wavelength calibration at KWS-2 is typically carried out using the Bragg reflections from silver behenate (AgBeh) powder (ARMAR Chemicals). The Bragg spacing of AgBeh is known to be *d* = 58.38 Å (Gilles *et al.*, 2000 ▸; Okabe *et al.*, 2007 ▸). AgBeh can also be used as reference material for determining the instrumental resolution parameters (Grillo, 2008 ▸) when Bragg peaks with several orders are observed at large angles (*Q* > 0.1 Å^−1^). In this range the uncertainty in *Q* is mainly governed by the wavelength spread Δλ/λ.

**Figure 10:**
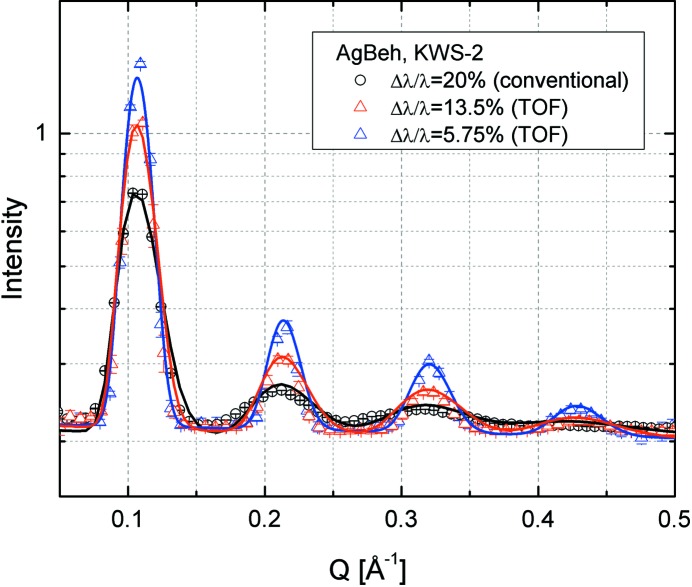
SANS data of the AgBeh reference sample measured at KWS-2 in conventional (black) and TOF (red and blue) modes for different Δλ/λ_aim_. Only data recorded in the central TOF channel are shown.

Fig. 10 ▸ shows scattering data of AgBeh collected at a very short detection distance *L*_D_ = 1.3 m with λ = 4.72 Å in conventional (Δλ/λ = 20%) and TOF modes. The chopper settings were chosen to correspond to the configurations (slit opening window) in Figs. S5 and S7. The chopper frequency was in these cases *f*_chopper_ = 35.4 and 41.2 Hz, while the expected wavelength spread was Δλ/λ_aim_ = 5.8 and 13.4%, respectively. Following a similar procedure to that used by Grillo (2008 ▸), the experimental curves were fitted simultaneously with fixed parameters, either known from SAXS measurements (peak position and width) or calculated (σ_geo_), and Δλ/λ as fitting parameter for each configuration. The model curves describing the experimental data delivered for the wavelength spread Δλ/λ the values 19.3, 13.1 and 7%, respectively, which are in good agreement with the expected results.

### 5.3. Opal   

**Figure 11:**
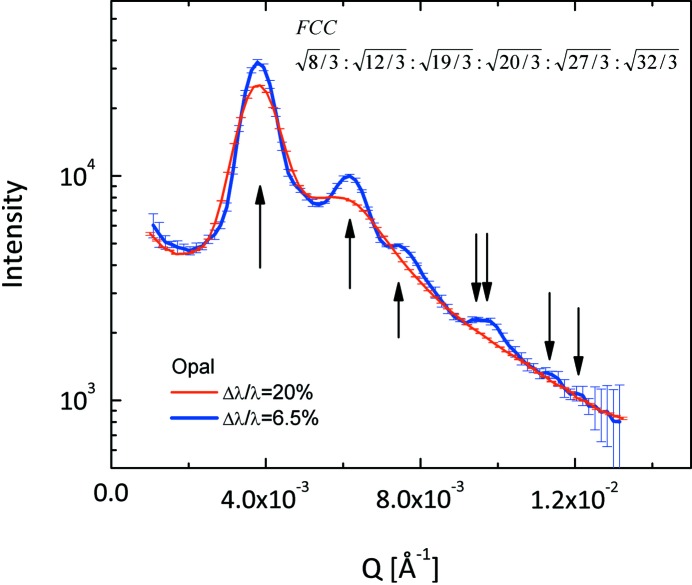
SANS curves of opal reference sample measured at KWS-2 in conventional (red) and TOF (blue) modes for different Δλ/λ_aim_. Only data recorded in the central TOF channel are shown. The arrows indicate the positions of the peaks observed in the TOF data, which denote an FCC arrangement of silica spheres in opal.

Fig. 11 ▸ shows the scattering curves measured on precious opal, a system which gives rise to intensity maxima at very small scattering angles due to Bragg reflections from the closest packed non-crystalline silica spheres (Graetsch & Ibel, 1997 ▸). The measurements were carried out with a neutron wavelength λ = 10 Å at a detection distance *L*_D_ = 20 m again in conventional (Δλ/λ = 20%) and TOF modes. In TOF mode the chopper configuration (slit opening width) was chosen as in Fig. 10 ▸ for a chopper frequency *f*_chopper_ = 9.7 Hz. While in conventional mode two broad peaks are revealed, in TOF mode up to seven peaks can be identified. The relation between the peak positions corresponds to a face-centred cubic (FCC) lattice characterizing the opal sample.

### 5.4. Diblock copolymer micelles   

**Figure 12:**
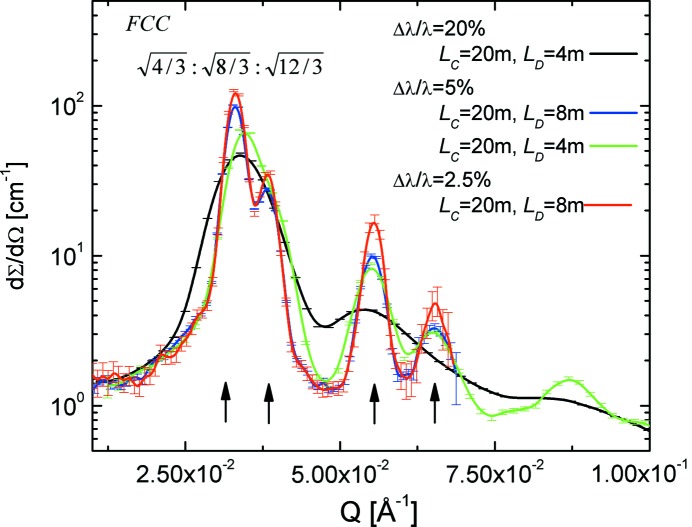
SANS curves of the C_28_H_57_–PEO5 polymer micelles in D_2_O measured at KWS-2 for different *L*_C_–*L*_D_ configurations in conventional (black) and TOF (red, blue and green) modes for different Δλ/λ_aim_. Only data recorded in the central TOF channel are shown.

The ordering structure of star-like C_28_H_57_–PEO5 polymer micelles in D_2_O was investigated at a high polymer volume fraction (12%) in conventional (Δλ/λ = 20%) and TOF mode (Fig. 12 ▸). Three broad peaks were observed in the data collected in conventional mode, while the high resolution revealed an underlying fine structure of the peaks that enables the explicit identification of the crystalline phase (FCC) formed by the micelles in solution.

## 6. Conclusion   

In this paper we have shown how the variation of the experimental resolution is performed at the high-intensity/extended *Q*-range SANS diffractometer KWS-2 by using a double-disc chopper with a variable opening slit window in concert with a velocity selector and data acquisition in TOF mode. The new option enables the possibility of tuning at will the wavelength resolution Δλ/λ within a broad range, from 20% (standard) down to 2%, in a convenient and safe way following pre-planned or spontaneous decisions taken during the experiment. The approach is of special value for the study of crystallizable soft-matter and biological systems or morphologies characterized by low polydispersity. This novel way to use the time-of-flight technique in conjunction with a traditional velocity selector represents an optimal solution considering the advanced goals of the experimentalists (adjustable wavelength resolution and manoeuvrability) and the severe predefined space conditions (tight beamline layout of KWS-2, lack of space, predefined selector environment – in the middle of the S-guide, increased safety requirements). Because of this, any other method capable of leading to the same result was dismissed from the very beginning.

## Supplementary Material

Supplementary Material - design, operation and pulse profile of the KWS-2 chopper . DOI: 10.1107/S1600576715019019/ge5015sup1.pdf

## Figures and Tables

**Figure 5:**
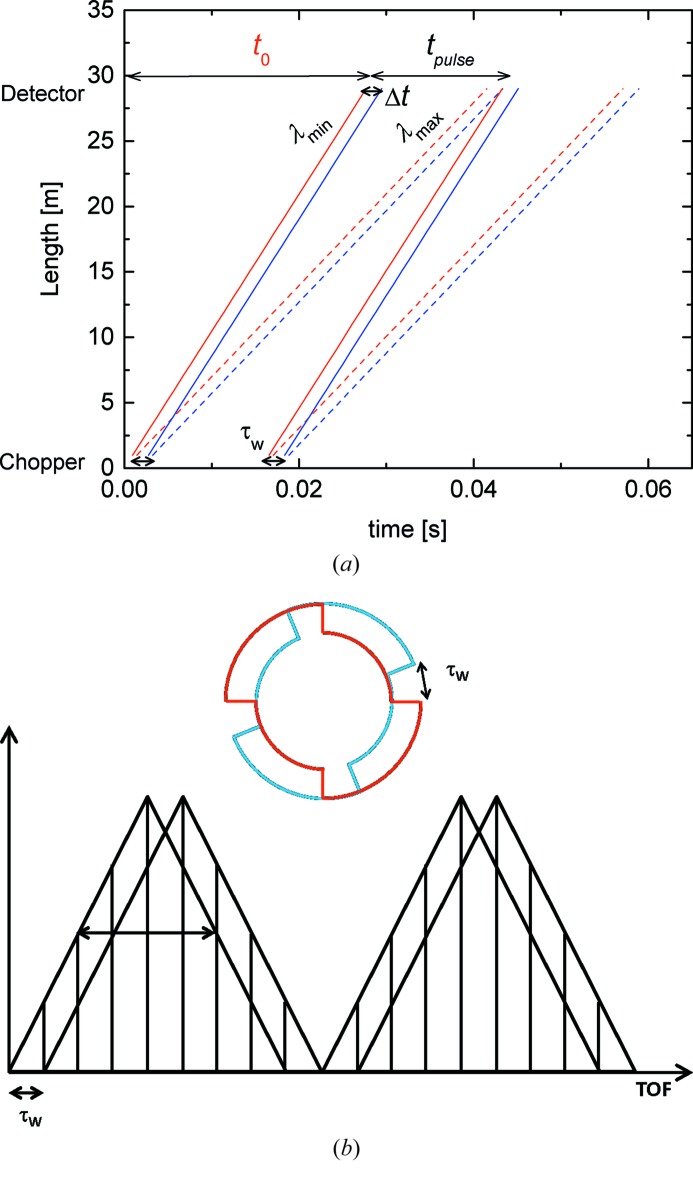
The same as in Fig. 4 ▸ for the setup corresponding to Δλ/λ_aim_ = 5%: *L*_D_ = 8 m, λ = 4.72 Å, *f*_chopper_ = 32.4 Hz, Δφ = 20°; τ_w_ = 0.0017 s. To reach the Δλ/λ_aim_ each pulse must be split into nine TOF slots.
