# Supplementary material for: Tuning the instrument resolution using chopper and time of flight at the small-angle neutron scattering diffractometer KWS-2
Source: J Appl Crystallogr. 2015 Nov 19;48(Pt 6):1849–59. doi: 10.1107/S1600576715019019 (PMC4665661; doi:10.1107/S1600576715019019)
Supplement: Supplementary file 1 [file j-48-01849-sup1.pdf]

JOURNAL OF APPLIED CRYSTALLOGRAPHY

Volume 48 (2015)

Supporting information for article:

Tuning the instrument resolution using chopper and TOF at the SANS diffractometer KWS-2

Aurel, Noémi Kinga, Stephan, Marko, Matthias, Johan, Matthias, Ralf, Romuald, Günter, Peter, Aristeidis, Vitaliy, Lutz, Henrich and Dieter

Tuning the instrument resolution using chopper and TOF at the SANS diffractometer KWS-2 – Supplementary material

Aurel Radulescu[a]*, Noémi Kinga Székely[a], Stephan Polachowski[b], Marko Leyendecker[b], Matthias Amann[c], Johan Buitenhuis[d], Matthias Drochner[b], Ralf Engels[b], Romuald Hanslik[b], Günter Kemmerling[b], Peter Lindner[e], Aristeidis Papagiannopoulos[f], Vitaliy Pipich[a], Lutz Willner[c], Henrich Frielinghaus[a] and Dieter Richter[c]

[a]Jülich Centre for Neutron Science (JCNS), Outstation at MLZ, Forschungszentrum Jülich GmbH, Lichtenbergstr. 1, Garching, 85747, Germany

[b]Zentralinstitut für Engineering, Elektronik und Analytik (ZEA), Forschungszentrum Jülich GmbH, Jülich, 52425, Germany

[c]Jülich Centre for Neutron Science (JCNS) and Institute for Complex Systems (ICS), Forschungszentrum Jülich GmbH, Jülich, 52425, Germany

[d]Institute for Complex Systems (ICS), Forschungszentrum Jülich GmbH, Jülich, 52425, Germany

[e] Institut Laue-Langevin, 71 Avenue des Martyrs, Grenoble, 38000, France

[f]Theoretical and Physical Chemistry Institute, National Hellenic Research Foundation, 48 Vass. Constantinou Ave., Athens, 11635, Greece

Correspondence email: a.radulescu@fz-juelich.de

Table S1 Summary of the quantities and parameters involved in the determination of the instrumental resolution for different experimental configurations routinely used at KWS-2; λ – neutron wavelength, $\Delta\lambda/\lambda$ –wavelength spread, L_D – detection length, L_C – collimation length, $X_{\min}$, $Y_{\min}$ – beam spot size (horizontal, vertical); all quantities and parameters are defined within the text; the calculations were done according to Eq. 2-4 and to Hammouda & Mildner (2007), for the size of the source and sample rectangular apertures 30mmx30mm and 10mmx10mm, respectively (R_E=17.3mm and R_S=5.78mm, after the conversion of rectangular aperture on round aperture of the same area).

Parameter									
λ [Å]	4.72	5	5	5	5	5	5	10	10
$\Delta\lambda/\lambda$	0.2	0.2	0.2	0.2	0.05	0.05	0.05	0.2	0.05
$(\sigma_Q^2)_{\mathrm{wav}}$ [Å^{-2}]	0.0067	0.0067	0.0067	0.0067	0.0004	0.0004	0.0004	0.0067	0.0004
L_D [m]	1	4	8	20	4	8	20	20	20
L_C [m]	4	4	8	20	20	20	20	20	20
$(\sigma_{Qx}^2)_{\mathrm{geo}}$ [Å^{-2}]	7.5E-6	1.24E-5	3.11E-6	4.97E-7	3.59E-6	2.59E-7	4.97E-7	1.24E-7	1.24E-7
$(\sigma_{Qy}^2)_{\mathrm{geo}}$ [Å^{-2}]	7.5E-6	1.24E-5	3.11E-6	5.06E-7	3.59E-6	2.59E-7	4.98E-7	1.63E-7	1.26E-7
X_{min} [cm]	1.505	3.236	3.236	3.236	1.389	1.852	3.23	3.23	3.23
Y_{min} [cm]	1.506	3.245	3.275	3.48	1.397	1.868	3.29	4.22	3.48
Q_{min}^x [Å^{-1}]	0.0189	0.010	5.1E-3	2.03E-3	4.36E-3	2.90E-3	2.03E-3	1.0E-3	1.0E-3
Q_{min}^y [Å^{-1}]	0.0189	0.010	5.1E-3	2.2E-3	4.38E-3	2.93E-3	2.07E-3	1.3E-3	1.1E-3

1. The design and operation of the KWS-2 chopper

The chopper system was designed, produced and installed at KWS-2 by the Central Institute for Engineering, Electronics and Analytics – Engineering and Technology (ZEA-1) of Research Centre Jülich GmbH.

The chopper contains two identical discs which are driven independently of each other by two rotor shafts. Both discs rotate counter-clockwise when viewed from the neutron source in the direction of the sample. The rotation frequency is the same for both discs and can be adjusted within a wide range, between 5 and 200 Hz. The discs have a diameter of 620mm and are placed at 5cm from each other. Each disc has two 90° windows with a depth of 65mm (Fig. S1). The rims of the discs out of the window area are coated over a wider range than the height of the neutron guide with ^{10}B containing epoxy paint disposed in a layer of a few tenth of a mm on each of the disc faces (Fig. S1). The content of ^{10}B is of 32 mg/cm^2, which enables a neutron transmission as low as 10^{-6}.

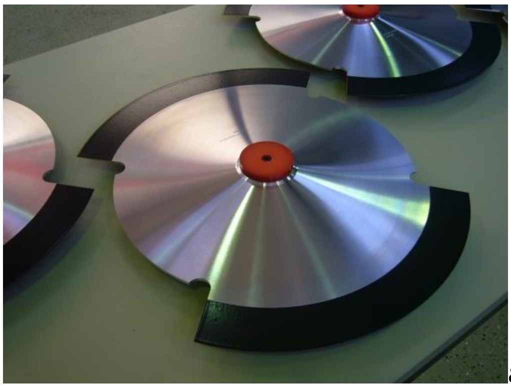

a)

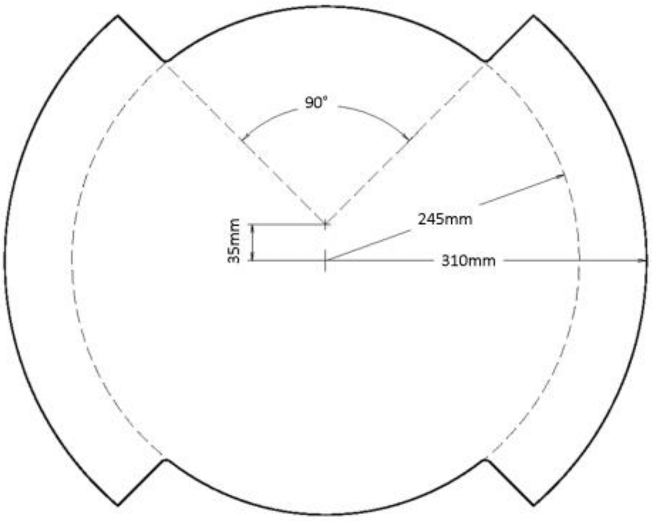

b)

Figure S1 The discs (a) and the slit geometry (b) of the KWS-2 chopper.

The chopper system is located at 0.7 m in front of the beginning of the collimation system. The chopper-to-detector distance L_{TOF} can be thus varied between 21.7 m and 40.7m. The whole system is placed in a stainless steel housing consisting of two compartments (Fig. S2a-d). The lower compartment contains the last segment of the "S"-guide, which is equipped with 2 slits and a thicker base to allow the discs to block and open the guide over its entire height. The special operating vacuum conditions of the chopper, of about $2x10^{-4}$ mbar, are achieved over the entire length of the second half of the "S"-guide, which is separated from the other vacuum ranges of the instrument by Al-windows. The upper compartment enables an easy removal of the discs (together with the rotors) in case of maintenance or repair.

The chopper housing is placed inside of a lead castle with 10cm thick walls covered on the inner faces with a 0.5cm thick layer of B_4C plastic, which ensures that the value of the γ and neutron background around the chopper stays within the accepted limits.

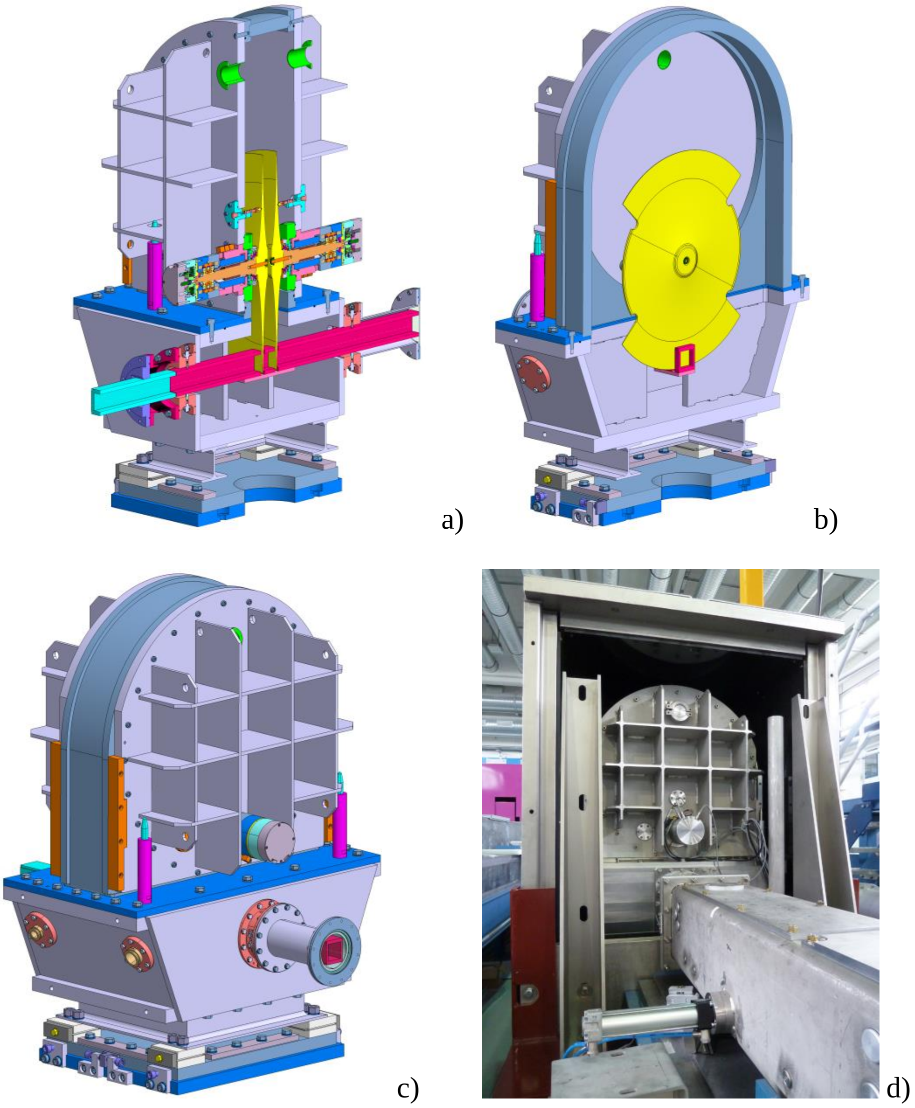

Figure S2 Design sketch (a-c) and view of the installed chopper, chopper housing and shielding (d) at KWS-2; the diametrically opposed 90°-windows and the ^{10}B-resin covered rims open and close the neutron guide over its entire height.

The opening and closing of neutron guide is schematically shown in Fig. S3a-d. The slightly larger blue circle indicates the position of the fixed pickup sensor on the housing (visible also in Fig. S2a), while the small black dot indicates the position of the pickup magnet, which is glued onto the disc. When the magnet passes the pickup sensor, the disc must rotate further 45 ° for the opening of the neutron guide to begin simultaneously over the full height (Fig. S3a). At 56.12 ° the guide is fully open (Fig. S3b). The closing of the guide starts at 123.88 ° (Fig. S3c) and ends at 135 ° (Fig. S3d).

In order to open and close the guide each disc takes 11.12 °. Fig. S4 illustrates the entire process in terms of the neutron intensity as a function of disc position. The pickup signal is also shown symbolically: the rising edge of the signal indicates the time when the magnetic pickup passes through the magnetic pickup sensor, which triggers the TOF detection electronics.

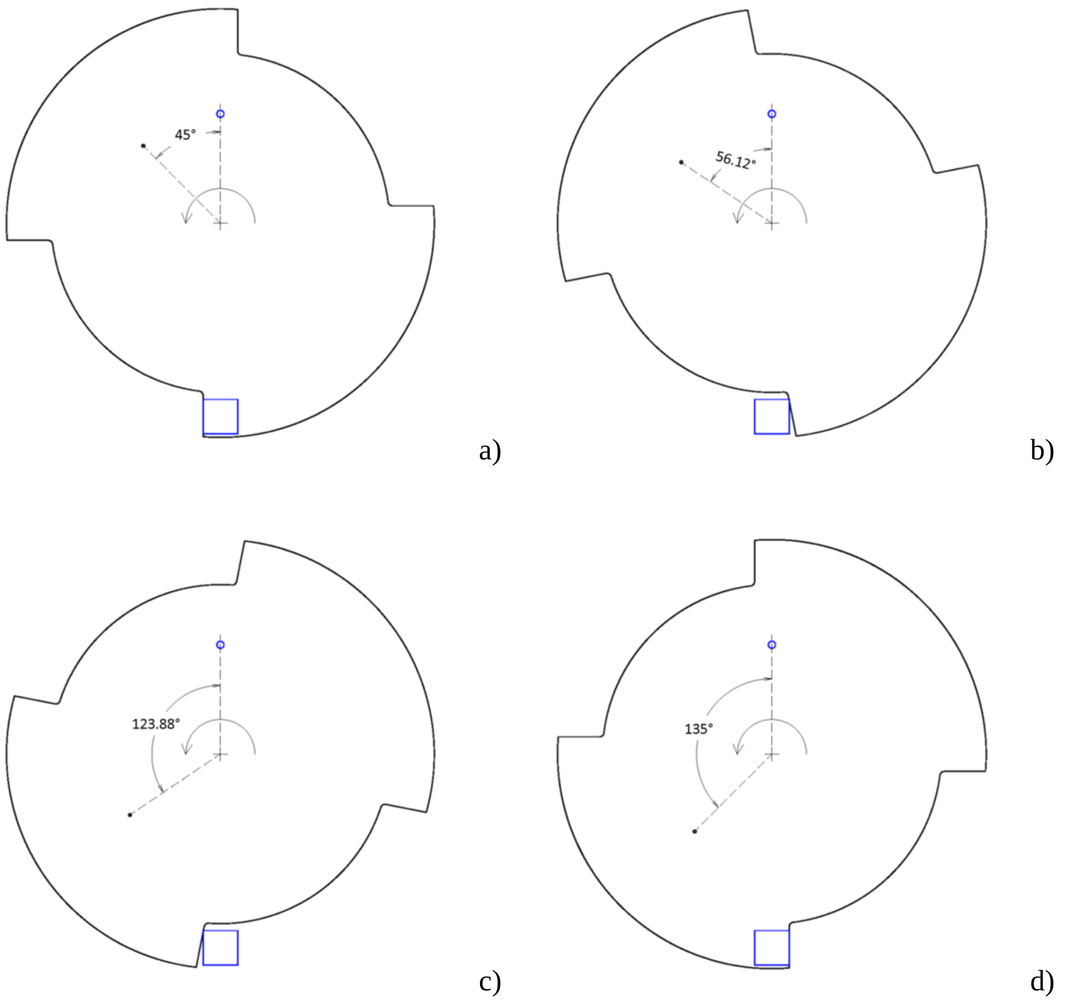

Figure S3 The relation between opening (a, b) and closing (c, d) of neutron guide (blue rectangle) and the relative position of the pickup-magnet (black dot) to pickup-sensor (blue circle); the viewing direction is in the neutron flight direction.

The chopper can be theoretically regarded as a single-disc with variable slit opening: a disc opens the neutron guide and the other closes it again. Essential in this process is only the difference between the two reference phases and not their absolute values. The phase difference $\Delta\Theta$ determines which disc opens the guide and which closes it again and which is the angular range (or time) over which the guide is opened or closed. The phase measurement is thus a measurement of time: the actual time

relates to the actual phase and chopper frequency through t [ms] = Θ_{is} [°] / (360° * $f_{chopper}$ [Hz]). The drive control of the chopper ensures a phase control precision within less than 0.01°.

Fig. S5 illustrates the relationship between the chopper phases and the master pulse for a situation when the phases are of 45 ° and 90 ° for the 1st disc and 2nd disc, respectively. In addition, the neutron flux is represented symbolically. Practically, phase settings like 45° / 90°, 10° / 55° or 200° / 245° lead to absolutely identical pulse structures. Only the relative position of the master pulse and pickup pulses would change in these cases. Therefore, the TOF detection electronics can be triggered with one of the two pick-up signals.

At a phase difference of $\Delta\Theta$=67.76 ° the resulting slit width ($\Delta\varphi$=90°-$\Delta\Theta$) corresponds to the width of the neutron guide. Fig. S6 illustrates the position of the chopper discs for this setting. Fig. S7 depicts the situation in temporal evolution. Again, only the phase difference decides. The phases 45° / 112.76° are shown, but the same result would be obtained also for example for combinations like 100° / 167.76° or 200° / 267.76°.

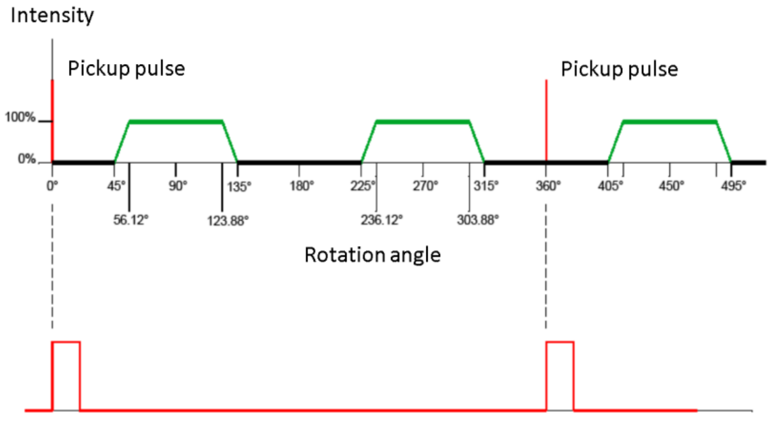

Figure S4 The same processes as in Fig. 3 presented in terms of the neutron intensity as a function of disc position (disc opening and pickup signal) in linear time sequence.

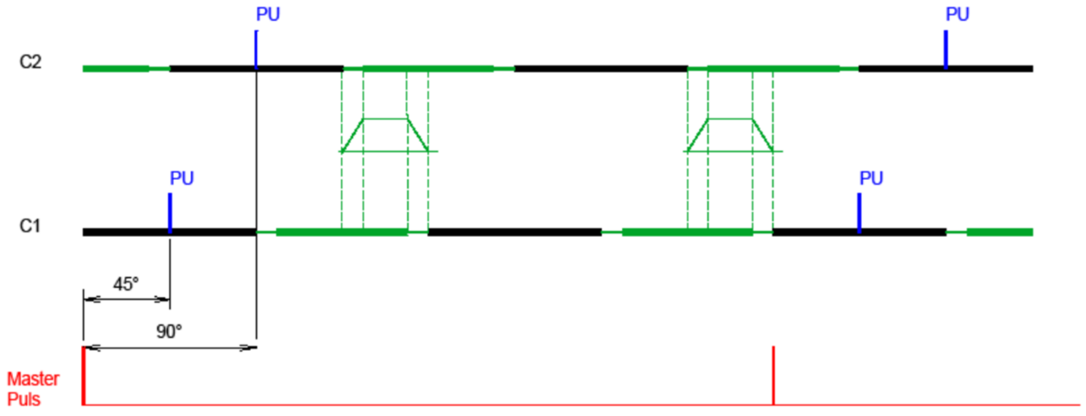

Figure S5 Schematic presentation of the relationship between the phase difference of chopper discs and neutron intensity at the phase setting 45°/90° in linear time sequence.

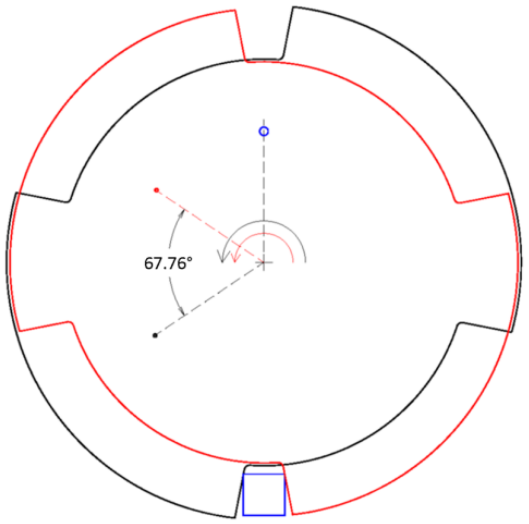

Figure S6 The neutron guide (blue) is fully opened twice per cycle for a short time when the two chopper discs have a phase difference of $\Delta\Theta$=67.76° to each other; this corresponds to an opening slit window of $\Delta\varphi$=22.24°.

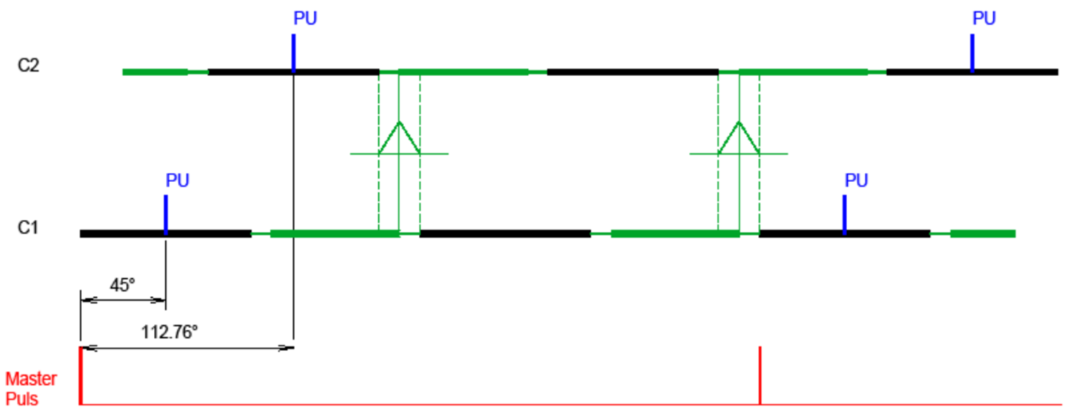

Figure S7 The neutron intensity in linear time sequence for a phase setting corresponding to the configuration shown in Fig. 6.

2. Merging of the two identical pulses

Fig. S8 shows the direct beam intensity on the detector as it was collected in TOF mode for λ=4.72Å, L_D=1.3m, L_C=20m and four values of the aimed wavelength resolution (in the central time slot), $\Delta\lambda/\lambda_{aim}$= 20%, 10%, 5% and 2.5%. The slit opening window $\Delta\varphi$ was 60°, 36°, 20° and 10.6°, while the chopper frequency was set to 32.0Hz, 37.9Hz, 42.13Hz and 44.6Hz, respectively. The intensity distribution in the Fig. S8b shows a rather trapezoidal profile in the case of $\Delta\lambda/\lambda_{aim}$=20% and 10%, while for $\Delta\lambda/\lambda_{aim}$=5% and 2.5% the profile is triangular. These two profiles are similar to those schematically shown in Figs. 5S and 7S, corresponding to a slit with either larger than the guide width (Fig. S5) or equal to it (Fig. 7S).

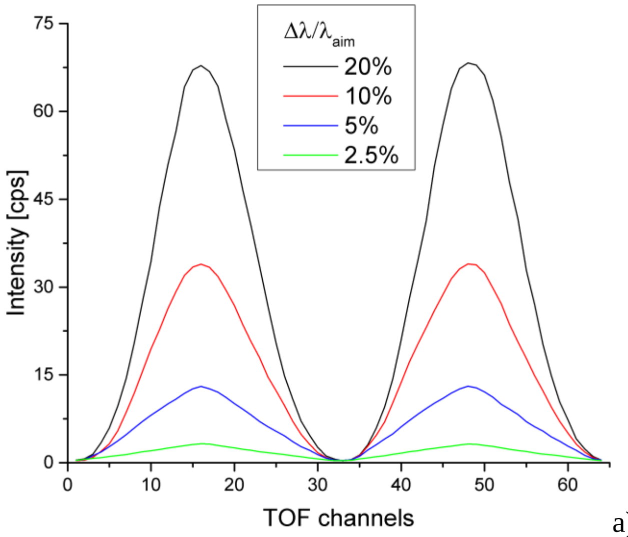

a)

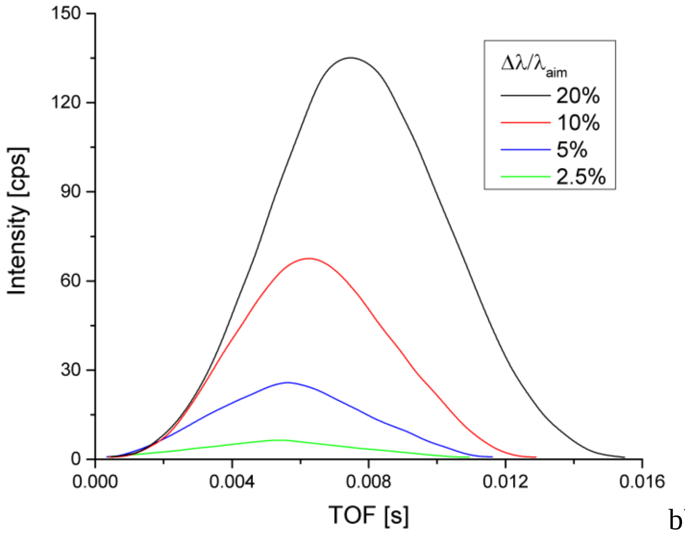

b)

Figure S8 The direct beam intensity (expressed in counts per second) on the detector (L_D=1.3m) measured for λ=4.72Å in TOF mode for different chopper settings corresponding to $\Delta\lambda/\lambda_{aim}$=20%, 10%, 5% and 2.5%: intensity distribution over the TOF channels (a) and as a function of TOF, after merging the two pulses into a single one.
